# Development of a tetra-primer ARMS–PCR for identification of sika and red deer and their hybrids

**DOI:** 10.1007/s44211-023-00405-6

**Published:** 2023-08-17

**Authors:** Yu Ke-xin, Chen Xiang, Hu Qing-qing, Yao Yi-an, Wang Xiao-ming, Xu Ai-chun, Ge Jian, Guan Feng

**Affiliations:** 1 College of Life Sciences, China Jiliang University, Hangzhou, 310018 China; 2 Zhoushan Institute for Food and Drug Inspection and Testing, Zhoushan, 316021 China

**Keywords:** Sika deer, Red deer, Hybrid, Tetra-primer ARMS–PCR, Species identification

## Abstract

Accurate identification of deer-derived components is significant in food and drug authenticity. Over the years, several methods have been developed to authenticate these products; however, identifying whether female deer products are hybrids is challenging. In this study, the zinc finger protein X-linked (*ZFX*) gene sequences of sika deer (*Cervus nippon*), red deer (*Cervus elaphus*) and their hybrid offspring were amplified and sequenced, the X221 and X428 species-specific single nucleotide polymorphisms (SNP) loci were verified, and a tetra-primer amplification refractory mutation system (T-ARMS–PCR) assay was developed to identify the parent-of-origin of female sika deer, red deer, and their hybrid deer. The T-ARMS–PCR developed based on the X221 locus could identify sika deer, red deer, and their hybrid offspring according to the presence or absence of PCR product sizes of 486 bp, 352 bp, and 179 bp, respectively, just as X428 locus could identify sika deer, red deer, and their hybrid offspring according to the presence or absence of PCR product sizes of 549 bp, 213 bp, and 383 bp, respectively. Forty products labeled deer-derived ingredients randomly purchased were tested using this assay, and the results showed that the identification results based on the two SNP loci were utterly consistent with the actual sources. In addition, this method was found to be accurate, simple, convenient, and with high specificity, thus providing an essential technical reference for deer product species identification. It is also an important supplement to the identification methods of the original ingredients of existing deer products.

**Graphical abstract:**

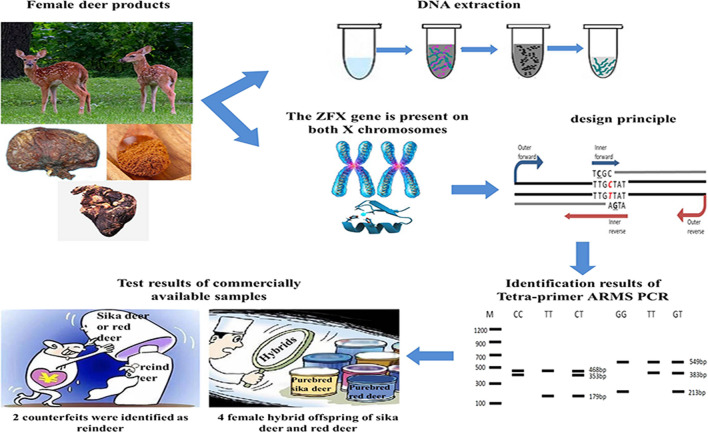

## Introduction

Accurate species identification and parental traceability are fundamental requirements for food and drug authenticity [1–6]. Deer are hoofed ruminant mammals that belong to Cervidae, which includes 10 genera and 17 species in China. The majority of these species are usually used in folk medicine; e.g., sika deer (*Cervus nippon*) and red deer (*Cervus elaphus*) can be used in Chinese traditional medicine [7]. Sika deer are listed with tonic properties in many ancient Chinese pharmaceutical monographs and have been bred for deer antlers. Furthermore, deer antler, also called “Lurong” in China, is produced from male deer and is widely sold in many Asian countries. However, it had been proved that amino acid content, inorganic elements and other nutritional compositions of velvet antler had some differences between cross-hybrid and purebred sika deer [8]. Consequently, there is also a significant difference in the sale price between hybrid sika deer antlers and pure sika deer antlers, resulting in the frequent emergence of adulterated deer products in the market. These products include venison, blood, heart (powder), fetus (powder), tendons and tails, etc. However, it is difficult to accurately identify the gender and species of these products only by morphological identification, and there is no report on the identification of the parental lineage of female deer hybrids.

At present, distinguishing and identifying sika and red deer is considered to be the key content for identifying deer origin. Among available methods, DNA-based molecular technology can accomplish the source identification of antler authenticity and hybridization [9–12]. For example, assays based on assessing specific DNA sequences from mitochondrial DNA (mt DNA) or X- and Y-chromosome genes can be used to identify sika deer and red deer from other animals [13–16]. Mt DNA was often selected as target genes for species identification. For instance, cytochrome C oxydase I (*COI*) and cytochrome B (*CytB*) had been used to identify deer-derived components and further to determine deer species [17]. In addition, the sex determining region of Y chromosome gene (*SRY*) was utilized in conjunction with the mitochondrial gene for further parental origin identification of velvet antlers. Wei et al*.* [18] combined *COI* with *SRY* genes to identify the antlers of sika deer, red deer, and their hybrid. Yet, this approach is not suitable for female deer hybrid identification. Based on molecular marker technology, single nucleotide polymorphism (SNP) was regarded as the third molecular marker, it has also been widely used in the latest years for species identification, genetic diversity analysis of biological populations, and molecular marker-assisted selection [19–22]. Ba et al. [23] screened the whole genome to search for SNP locus in 30 distinct hybrid progenies, and suggested that there were 2015 diagnostic SNP loci could be used for the identification of sika deer, red deer and hybrid progenies. However, there was no literature report on the use of these SNPs to identify deer species. In addition, it was reported that the random amplified DNA polymorphism PCR assay (RAPD–PCR) had been used to discriminate the hybrid deer species based on mt DNA [24, 25]. Nevertheless, there still has a challenge in distinguishing purebred sika deer and hybrid deer using these mentioned methods. The existing DNA typing approaches used to identify hybrid deer species were confined to only identify male deer products, such as antlers and whips. Thus, in this study, the *ZFX* gene fragment located on the two X chromosomes of female deer was selected and sequenced to search for species-specific variations and further identify the deer species. The verified specific SNPs were used to develop a T-ARMS–PCR for identifying the parental origin of crossbred female deer based on the differences between the two X chromosomes, thereby resolving the current challenges in determining the parental origin of female deer hybrid.

## Materials and methods

### Preparation of samples

To develop T-ARMS–PCR assay, fifteen female sika and red deer muscle tissue and blood samples were collected from Zhejiang Tianmu Mountain Nature Reserve, the Wildlife Conservation Station of Qinghai Province, and the Jinnao Farm in Shuangyang Deer Township, Heilongjiang Province, from 2019 to 2020. The samples included five sika deer, five red deer, and five of their hybrid offspring.

### DNA extraction and quality test

The genomic DNA of each sample was extracted using an Animal Tissue Genomic DNA Extraction Kit (Hangzhou Simgen biochemical reagent Development Co., Ltd.) according to the manufacturer’s instructions. This kit can be used for DNA extraction from many tissues, including cells, blood, muscle, bone and hair; the process is adjusted according to the corresponding tissues used. All the samples were performed in duplicate, after which the higher quality data were selected for the next use. The extracted DNA was assayed using NanoDrop 2000 (Thermo Fisher Scientific) to determine its purity and concentration based on A260/A280 ratios. The instruments were calibrated using Tris–EDTA buffer solution (DNA TE buffer, Hangzhou Simgen biochemical reagent Development Co., Ltd.) at room temperature.

### Amplification and sequence data analysis of *ZFX* gene

The primers used for amplifying the *ZFX* gene fragment were designed according to the *ZFX* gene sequence of red deer [26]. The *ZFX* gene sequences of red deer (GenBank NO. KP 257294.1, CM008041.1:c153615887–153602265) and cattle (*Bos taurus*, GenBank NO. NC_037357.1) were compared, and the variations were selected as the target amplification fragment. Theoretically, the expected amplification region using the designed primers covers the variations between the two species. The primers were synthesized by Hangzhou Tsingke Biotechnology Co., Ltd., and the sequences are as follows:

Forward primer (ZFX1F): 5′-CCACAAGAACCAAACTCATT-3′,

Reverse primer (ZFX1R): 5′-TTGATAACTTCAGGGCAAG-3′.

The *ZFX* gene fragment was amplified from sika deer, red deer and their hybrid progenies using a basic 20 μL PCR system, respectively. The PCR conditions were as follows: 5 min at 95 ℃, followed by 35 cycles of 30 s at 94 ℃, 40 s at 55 ℃ and 2 min at 72 ℃; post-amplification for 10 min at 72 ℃, and the amplified product was stored at 4 ℃. After 1.5 w% agarose gel electrophoresis, the positive PCR products were taken to Tsingke Biotechnology Co., Ltd for Sanger sequencing.

Clustal X (version 2.1) software was used for sequence alignment, and the SNP locus was further aligned with the database in GenBank from red deer and sika deer.

### Development of a T-ARMS–PCR assay targeting two SNP loci

**Fig. 1:**
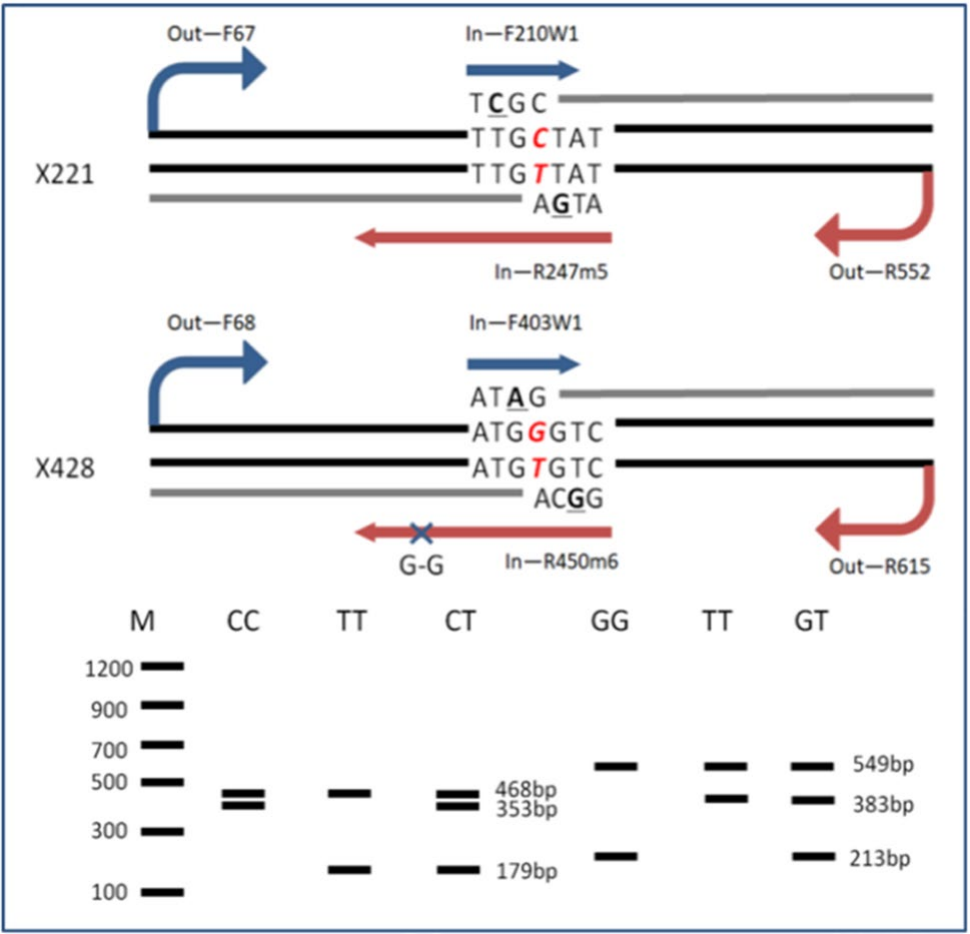
Schematic of T-ARMS–PCR primer design. The X221 locus is “CT” genotype, with “CC” representing the homozygote of sika deer and producing 468 bp and 353 bp. “TT” for the homozygote of red deer produces 468 bp and 179 bp. “CT” for the female hybrid offspring of sika and red deer and displays the three bands at the same time. The X428 locus is “GT” genotype, with “GG” representing the homozygote of sika deer and producing 549 bp and 213 bp. “TT” for the homozygote of red deer and producing 549 bp and 383 bp. “GT” represents the female hybrid offspring of sika and red deer and displays the three bands at the same time. “G–G” is a weak base mismatch added at the 5'-end of the In-R450m6. Underlined letters represent mismatched bases

Two species-specific SNP loci [X221 (C–T) and X428 (G–T)] were verified in the *ZFX* gene among red deer and sika deer, and they were used as DNA targets to design T-ARMS–PCR primers for distinguishing sika deer, red deer and their hybrid progenies, which appeared heterozygous at the two corresponding loci. Two primer sets for T-ARMS–PCR were designed, and the PCR product sizes were expected to be between 150 and 600 bp. T-ARMS–PCR primers were designed according to the protocol proposed by Ye et al*.* [27], and all mismatched bases were designed to make up for the lack of mismatch strength of the original primer weak mismatches [27, 28]. The mismatched base is shown in Table 1. The principle of primer design is illustrated in Fig. 1.

**Table 1:** Primer sequences for detection of mutations at X221 and X428 loci in T-ARMS–PCR

SNP	Primer name	Primer sequence (3′–5′)	Product size (bp)
X221	Out-F67	GCTGATGCTACACACACGGAT	
	In-F210w1	AGATGTTATCGAGGACGT**C**GC	486
	In-R247m5	TCTGCACATTGAACATCTTCTAT**G**A	w352
	Out-R552	TTCTGAGGCACAATCTGCTACC	m179
X428	Out-F68	GCTGATGCTACACACACGGATG	
	In-F403w1	GTCTTGACGAGTGAATCCATACAT**A**G	549
	In-R450m6	TCAA**G**GTGTCCAATGTCAG**G**CA	w213
	Out-615	CTCACAGTTGCCTTTGTCATCCT	m383

“w” included name represents the inner forward primer and “m” represents the inner reverse primer. Underlined letters represent mismatched bases

### Amplification of T-ARMS–PCR

Amplification was performed on the PTC-200 (Bio-Rad Laboratories, Inc.) instrument based on the optimized results, and two amplification procedures were set for two deer *ZFX* SNP loci X221 and X428.

### Locus X221 detection system

The reaction of the locus X221 T-ARMS–PCR was performed in a total volume of 20 μL, containing 2 μL of 10 × buffer (Thermo Fisher Scientific), 1.6 μL of dNTPs mixture (25 mM, Thermo Fisher Scientific), 2 μL of MgCl_2_ (25 mM, Thermo Fisher Scientific), 0.4 μL of outer forward primer Out-F67 (10 mM), 0.6 μL of inner forward primer In-F201w (10 mM), 0.6 μL of inner reverse primer In-R247m5 (10 mM), 0.4 μL of the outer reverse primer Out-R552 (10 mM), 0.4 μL of *Taq* DNA polymerase (5 U/μL, Thermo Fisher Scientific), 3 μL of DNA template, and 9 μL of ddH_2_O. The PCR procedure consisted of initial denaturation at 95 ℃ for 5 min, followed by 32 cycles of denaturation at 94 ℃ for 35 s, extension at 62 ℃ for 40 s and annealing at 72 ℃ for 1 min; final extension was at 72 ℃ for 10 min and cooling 4 ℃ at last.

### Locus X428 detection system

The reaction of the locus X428 T-ARMS–PCR was performed in a total volume of 20 μL, containing 2 μL of 10 × buffer (Thermo Fisher Scientific), 1.6 μL of dNTPs mixture (25 mM, Thermo Fisher Scientific), 2 μL of MgCl_2_ (25 mM, Thermo Fisher Scientific), 0.5 μL of outer forward primer Out-F68 (10 mM), 0.5 μL of inner forward primer In-F403w1 (10 mM), 0.8 μL of inner reverse primer In-R450m6 (10 mM), 0.5 μL of outer reverse primer Out-R615 (10 mM), 0.4 μL of *Taq* DNA polymerase (5 U/μL, Thermo Fisher Scientific), 3 μL of DNA template, and 8.7 μL of ddH_2_O. The PCR procedure consisted of initial denaturation at 95 ℃ for 5 min, followed by 35 cycles of denaturation at 94 ℃ for 35 s, extension at 61 ℃ for 40 s and annealing at 72 ℃ for 1 min; and final extension at 72 ℃ for 10 min and cooling 4 ℃ at last.

The PCR product was examined by 2 w% agarose gel electrophoresis (Shanghai Sangon Biotech Co., Ltd.) and contained 1/10000 nucleic acid dye Gelred (BBI Life Sciences Corporation Co., Ltd.).

### Verification of the method in real case samples

A total of 40 servings of commercially available deer products were purchased from supermarkets, the internet and pharmacy, including 8 copies of venison, deer fetus powder, deer heart (powder), and deer blood of each type. These products are the most common deer-derived products besides deer antlers, and none of them can be judged by appearance.

Briefly, genomic DNA was extracted from all the samples according to the above method, after which the amelogenin gene (*AMEL*) gene sequence was amplified for sex identification [29]. The species identification was determined using DNA barcoding *COI* sequence and *SRY* gene as the targets if the deer product was derived from a male. However, the species identification was determined using this T-ARMS–PCR if the deer product was derived from a female.

Two T-ARMS–PCR systems for X221 and X428 loci were used to identify the authenticity and parental provenance of 36 commercial deer products after sex identification. The aim of the analysis was to authenticate the species declared on the product label. The labels were carefully inspected and compared with the obtained results of the analysis, which were associated either with correct labeling or mislabeling.

## Results

### DNA quality

The extracted DNA was assayed using NanoDrop 2000 to determine its purity and concentration. The tested results showed that the concentrations ranged from 40 to 230 ng/μL, the mean concentration was 86.4 ± 20.65 ng/μL, and the purity A260/A280 was 1.8–2.1; the ideal value for pure DNA samples was in the range of 1.7–2.0 and 1.8 was regarded as the optimal value [30]. In addition, higher values may be indicative of the presence of residual protein, while lower values can indicate very low DNA concentrations. These results suggested the presence of residual protein in all the DNA samples but met the need for further PCR analysis [31]. The DNA samples were diluted to a final concentration of 20 ng/μL and stored at 4 ℃ for later use.

### Amplification and sequence of *ZFX* gene

**Fig. 2:**
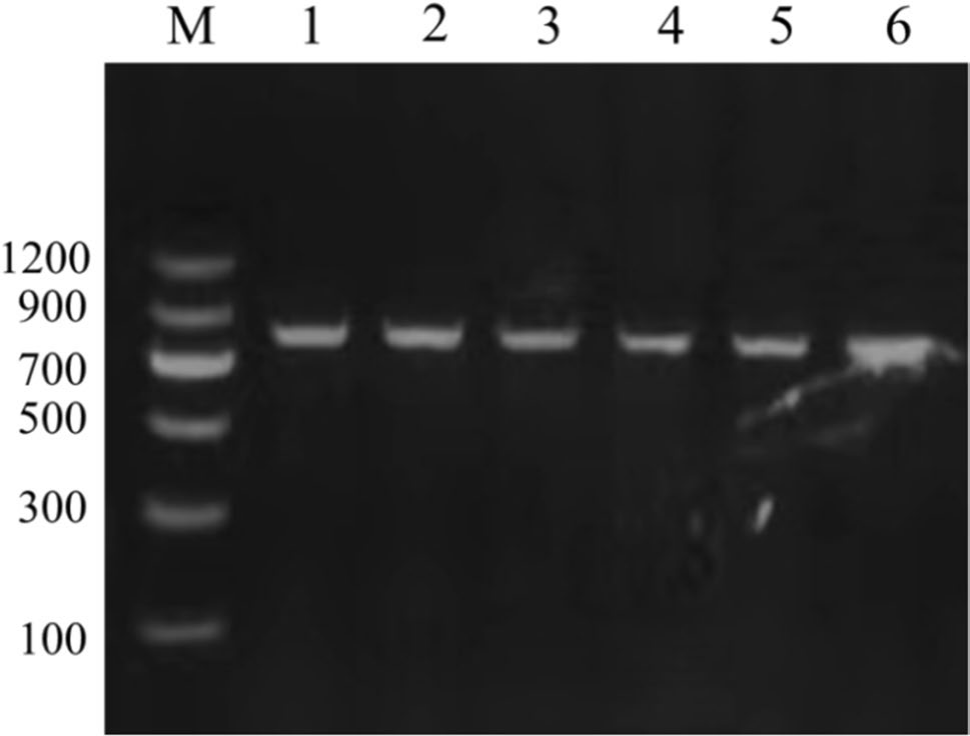
Results of *ZFX* gene amplification. Molecular size marker 100–1200 bp (M), lanes 1–2 = sika deer; 3–4 = red deer; 5–6 = Hybrid offspring of sika deer × red deer

**Fig. 3:**
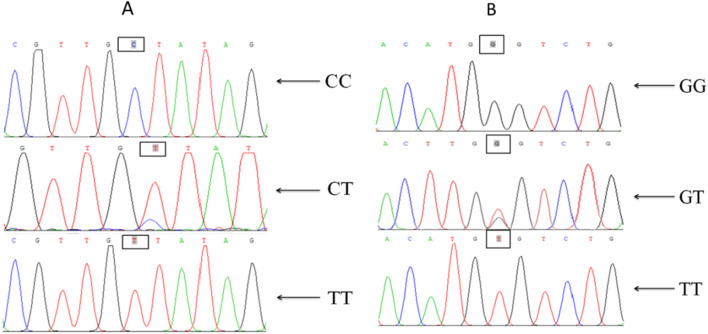
Sequencing results of X221 and X428 loci in *ZFX* gene.** A** The sequencing result maps of the X221 locus; genotype “CC” is specific for sika deer, genotype “TT” is specific for red deer, and genotype “CT” is specific for hybrid offspring of sika deer and red deer. **B** The sequencing result maps of X428 locus; genotype “GG” is specific for sika deer, genotype “TT” is specific for red deer, and genotype “GT” is specific for hybrid offspring of sika deer and red deer

The PCR amplification product of the *ZFX* gene was detected by 1.5 w% agarose gel electrophoresis, and the results showed that the positive PCR product size was about 730 bp, which was according to the expected sizes (Fig. 2). The sequencing results were consistent with the sequencing peak diagram. Editseq (version 7.1.0) and MegAlign (version 7.1.0) softwares were used to edit, splice, and compare sequences of sika deer, red deer, and hybrid deer. The sequences demonstrated that the five sika deer individuals' sequences were identical. In addition, two species-specific single nucleotide polymorphic loci were verified between the two species, i.e., SNP X221 (C–T) and SNP X428 (G–T), which appeared heterozygous in hybrid offspring. The findings were identical to the hybrid deer sequencing results. The homozygous genotypes of sika deer were CC and GG at X221 (C–T) and X428 (G–T) loci, that of the red deer was TT genotype at the two loci, and the female hybrid offspring was CT and GT heterozygous genotypes, respectively. DNA sequencing results of X221 and X428 loci by Sanger sequencing are shown in Fig. 3.

### Amplification and typing results of T-ARMS–PCR

**Fig. 4:**
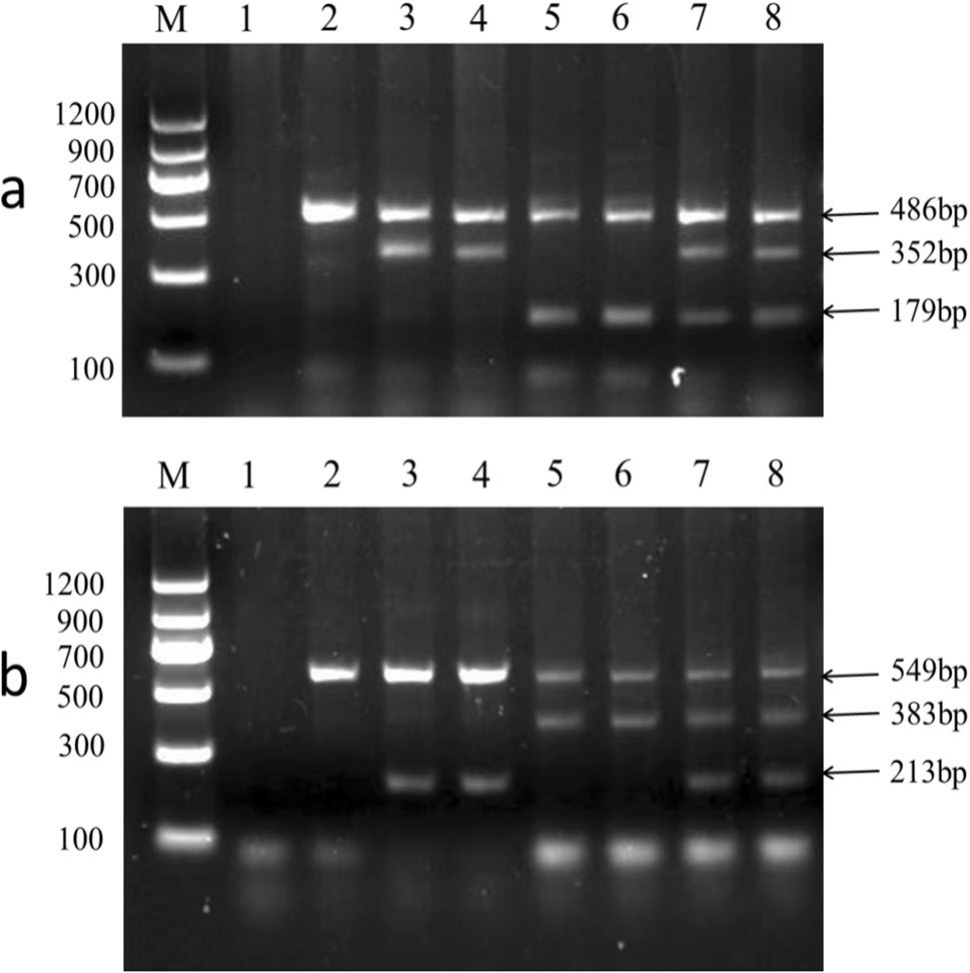
T-ARMS–PCR assays for X221 locus (**a**) and X428 locus (**b**). Molecular size marker 100–1200 bp (M), lane 1 = Negative control; 2 = reindeer (*Rangifer tarandus*); 3–4 = sika deer; 5–6 = red deer; 7–8 = Hybrid offspring of sika deer × red deer. **a** Typing result of X221 locus. **b** Typing result of X428 locus

Using the genomic DNA of sika deer, red deer, and their female hybrid offspring as templates, two groups of tetra-primer sets were employed for PCR amplification, and the PCR products were separated by 2 w% agarose gel electrophoresis. The electrophoresis results showed that the two control fragments of 486 bp and 549 bp could be amplified for X221 and X428 loci of sika deer and red deer, respectively. Two expected detection fragments were amplified simultaneously for two homozygous loci of sika deer, which were 468 bp and 352 bp or 549 bp and 213 bp corresponding to X221 and X428 loci, respectively. In addition, two fragments, 468 bp and 179 bp, or 549 bp and 383 bp, could also be amplified in homozygous red deer. Furthermore, electrophoresis results indicated that three bands could be amplified in the female hybrid offspring of sika and red deer, which were 486 bp, 352 bp and 179 bp at X221 locus or 549 bp, 213 bp and 383 bp at X428 locus (Fig 4). It is worth mentioning that when the electrophoresis results only had control products for 486 bp or 549 bp fragments, the sample source belonged to Cervidae animals except for sika deer and red deer; these PCR products could be used as a positive control. In theory, pure male sika deer also has the *ZFX* gene obtained from a maternal inheritance; the male individual has 468 bp and 352 bp or 549 bp and 213 bp at X221 and X428 loci, respectively. Furthermore, pure male red deer also has the *ZFX* gene obtained from its maternal inheritance, where the male individual has 468 bp and 179 bp or 549 bp and 383 bp, correspondingly at X221 and X428 loci. However, the genotypes of the male hybrid deer at X221 and X428 loci are determined by its mother and remain consistent, because the male hybrid deer only has one *ZFX* gene from the mother. In the later, it was proved that the detection results of some antler DNA samples were consistent with our speculated theoretical results (shown in the part of market samples identification results).

### Market samples identification results

**Fig. 5:**
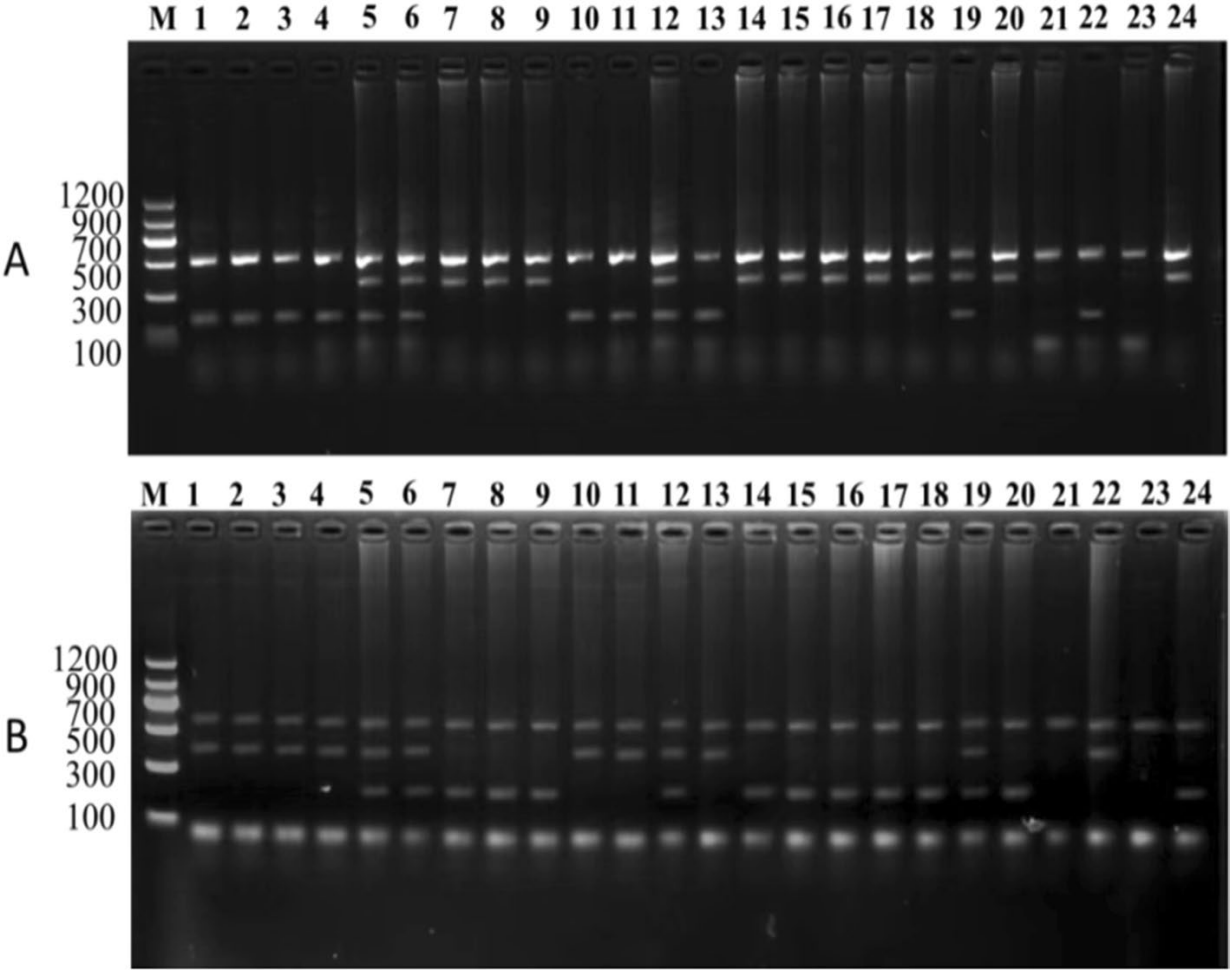
T-ARMS–PCR detection results of 24 market samples.** A** The identification results of the X221 locus. **B** Identification results of the X428 locus. Both identification results are consistent. Molecular size marker 100–1200 bp (M), 1–4 = red deer; 5–6 = Hybrid offspring of sika deer × red deer; 7–9 = sika deer; 10–11 and 13 = red deer; 12 = Hybrid offspring of sika deer × red deer; 14–18, 20, and 24 = sika deer; 19 = . Hybrid offspring of sika deer × red deer; 21 and 23 = reindeer (*Rangifer tarandus*); 22 = red deer

We randomly purchased 40 commercially available deer-derived products, including venison, deer fetus powder, deer heart (powder) and deer blood, which were not labeled with the deer gender. After sex identification, three blood and one meat sample were identified as male deer samples. Next, 36 genomic DNA samples were amplified using the above two primer sets. The PCR products were detected by 2 w% agarose gel electrophoresis, and the results containing 24 DNA samples were shown in Fig. 5. In this test, 4 female hybrid offspring of sika deer and red deer were identified, which were not consistent with the purebred sika deer tags. At the same time, 2 counterfeits were identified as reindeer (*Rangifer tarandus*) source ingredients. The rest of the results were consistent with the labels. The detection results of SNPs at X221 and X428 of *ZFX* gene were consistent with labeled as purebred sika deer, red deer, and their hybrid offspring. The identification results of both loci were the same and indicated that both loci could be used as diagnostic SNP loci for identifying sika deer, red deer and their hybrid progenies.

## Discussion

Deer is a traditional rare Chinese medicinal animal whose numerous body parts can be utilized as medicine. However, the value of deer products from purebred sika deer is generally considered higher than that of red deer and their hybrids, especially for velvet antlers [8]. The male deer is the sole producer of antlers and has been used for antler production for a long time without being slaughtered. Conversely, the older male deer are often slaughtered to obtain more products, even newborn cubs and mature female hybrid deer. Moreover, deer blood is often obtained from the male deer during antler cutting. Thus, it is impossible to determine the gender of multiple deer products in the market labeled as deer products solely based on appearance. Therefore, it is an important responsibility of the regulatory authorities to detect the species source of these deer products.

As is well-known, the sexuality of mammals is determined by their sex chromosomes. Females have XX chromosomes, and males possess XY chromosomes. The sex chromosomes contain essential genes for mammalian gender, including the *SRY* testis-determining gene, the zinc finger protein gene (*ZFX/Y*), the *AMEL*, the sex-determining region Y-box9 gene (*Sox9*), and the steroidogenic factor 1 gene (*SF1*), and others [32–34]. *ZFX/Y* genes are located on both X and Y chromosomes but are not homologous regions. During evolution, these two genes have inherited gender and species-specific information. The *ZFY* gene is more variable and active than the *ZFX* gene, and the *ZFX* gene is considered to be inactivated by the X chromosome [35]. Female gametes XX are genetically constituted of X chromosomes from each parent and carry *ZFX*-specific parental gene sequences. In this study, we screened and selected species-specific SNP locus in the *ZFX* gene sequences of sika deer and red deer and combined them with T-ARMS–PCR to identify females of hybrid. The results of this assay showed that the four-primer amplification blocked mutation system based on two SNP loci had adequate sensitivity, high efficiency and low cost.

The T-ARMS–PCR is a derived technique of conventional PCR for detecting various SNP sites [36–38]. The principle of the technique is that the *Taq* DNA polymerase lacks exonuclease activity at the 3′–5′ end. Accordingly, when a single base at the 3′ end is mismatched, the primer does not extend, whereas if it is complimentary, the PCR will continue [39]. Consequently, two specific internal primers with opposite extension directions and two positive control external primers can be designed for any SNP site [27, 40]. Based on the presence or absence and amplified product sizes of the four primers, it is possible to distinguish different genotypes. T-ARMS–PCR is suitable for identifying almost all single-base mutation loci and thus is widely used for cancer screening [41, 42], pathogen detection [22, 43–45], wild-type screening [40, 46, 47], food authenticity [17, 48], and genetic preference markers screening for economic animals [49–51], and other applications.

Currently, there are many PCR and derived methods used for species identification, including real-time PCR and multiplex PCR targeting mtDNA and nucleotide DNA, all of which can identify Cervidae animals, including sika deer, red deer, spotted deer, hog deer and mule deer, etc. [4, 17, 52, 53]. Nonetheless, the existing methods cannot solve the problem of species identification of hybrid deer, especially for females. The present study overcame a technical shortcoming in detecting deer-derived ingredients in food and medicinal products. Based on the species-specific SNP sites in the genomic sequences of sika deer and red deer, a T-ARMS–PCR technique was developed to determine whether or not a female deer was heterozygous and its paternal origin in current deer products. Since T-ARMS–PCR technique was developed, it has been widely used for SNP detection.

Following the T-ARMS–PCR principles, in addition to the mismatched site at the 3' end of the specific internal primer, a second or third mismatched base needs to be appropriately introduced to improve the specificity depending on the mismatch strength. The placement of the introduced mismatched site and the selection of its mismatch type are crucial for the effectiveness of the T-ARMS–PCR technique [27, 28]. Mismatched bases are typically located at the second or third-to-last position at the 3' end. For instance, Zhang et al*.* [54] introduced mismatched bases at the 3' end of the inner primer's penultimate position to increase the specificity. It has also been demonstrated that introducing mismatched bases in the fourth-to-last position tends to make the primers more specific and that placing mismatched bases in both positions 3 and 4 also make primer-specific amplification more accurate [55, 56]. In this assay, to improve the specificity of ARMS assays primers, C–A mismatched base was introduced at the 3' end of the inner forward primer (In-F201w1), and G–T mismatched base was introduced at the 3' end of the inner reverse primer (In-R247m5) for X221 (C–T) locus in accordance with the principles of mismatch strength and weakness. The design principle and mismatch base types of another pair of internal primers for the X428 (G–T) locus were the same as the X221 locus, and an additional G–G mismatched base was introduced at the 5' end of the In-R450m6 primer. We found that if only one mismatched base was located in the penultimate or the third position of the 3' end of the inner reverse primer, this primer would still has a significant mismatch, so a second mismatched base needed to be introduced. After PCR optimization and sequence alignment, it was found that the non-specific amplification of the primer could be interrupted by adding a weak mismatched base to the first 5 or 6 positions of the primer's positive 5' end. For this experiment, a mismatched base was introduced at a certain position at the 5' end to achieve the desired amplification effect. However, the applicability of this method to other SNP primers' design should be further investigated. Thus, to establish whether introducing a mismatched base or not and its corresponding location in the primer sequence had a crucial effect on the tetra-primer assay, we obtained the optimal solution by adjusting the mismatched base type and location of the introduced mismatches according to the mismatch type of the SNP site.

Optimizing the ratio between the four primers was also a key step in this assay. Since the inner primers contained several mismatched sites, while the outer primers were exact matched, the outer primer had higher amplification efficiency than the inner primer. This resulted in imbalanced amplification efficiency, which could only be resolved by increasing the concentration ratio of the internal primers to improve their amplification effectiveness. In addition, annealing temperature had a substantial effect on primer specificity. The specificity of the primer gradually increased as the annealing temperature increased. When Tm values were < 60 °C, the In-R247m5 primer at the X221 locus also mis-amplified the sika deer target fragment. However, when the temperature was increased to 62 °C, the primer only amplified the red deer target fragment and not the sika deer fragment. Consequently, the optimal annealing temperature was significant for optimizing the T-ARMS–PCR reaction conditions in this experiment.

To our knowledge, the present study is the first to propose the parentage detection method for female deer hybrids. The proposed method can validly identify female deer products source other than the antlers and the deer whips. This assay can provide pharmaceuticals and food regulatory agencies with technical support to detect and improve the effectiveness of food and herbal medicine market surveillance.

## Conclusions

In this study, the *ZFX* gene sequences of sika deer (*Cervus nippon*), red deer (*Cervus elaphus*) and their hybrid offspring were amplified and sequenced, and two fixed species-specific SNP loci were verified, which were X221 (C–T) and X428 (G–T), respectively. Combining the above SNP information with T-ARMS–PCR technology, the sika and red deer and their hybrid offspring could be effectively distinguished. The T-ARMS–PCR system developed based on the X221 locus could identify sika deer, red deer, and their hybrid offspring according to the presence or absence of PCR product of 486 bp, 352 bp, and 179 bp, just as the X428 locus could identify sika deer, red deer and their hybrid offspring according to the presence or absence of PCR product of 549 bp, 213 bp, and 383 bp, respectively. Randomly purchased 40 commercially available labeled deer-derived products were detected after sex identification, and the results showed that there were 4 female hybrid samples, which were not consistent with the product tags. At the same time, 2 counterfeits reindeer (*Rangifer tarandus*) source ingredients were detected. The rest of the results were consistent with the labels. The T-ARMS–PCR method was a simple and convenient assay, with high specificity, thus providing an essential technical reference for deer product species. The methods can be used to validly identify the source of female deer products other than the antlers and the deer whips. It is also an important supplement to the identification methods of the original ingredients of existing deer products.

## Data Availability

All the data used in the manuscript is available in the tables and figures. The datasets generated during and/or analysed during the current study are available from the corresponding author on reasonable request.
